# Polymorphisms in the Mitochondrial Genome Are Associated With Bullous Pemphigoid in Germans

**DOI:** 10.3389/fimmu.2019.02200

**Published:** 2019-11-22

**Authors:** Juliane Russlies, Anke Fähnrich, Mareike Witte, Junping Yin, Sandrine Benoit, Regine Gläser, Claudia Günter, Rüdiger Eming, Jeanette Erdmann, Damian Gola, Yask Gupta, Maike Marleen Holtsche, Johannes S. Kern, Inke R. König, Dimitra Kiritsi, Wolfgang Lieb, Christian D. Sadik, Miklós Sárdy, Franziska Schauer, Nina van Beek, Anke Weidinger, Margitta Worm, Detlef Zillikens, Enno Schmidt, Hauke Busch, Saleh M. Ibrahim, Misa Hirose

**Affiliations:** ^1^Luebeck Institute of Experimental Dermatology, University of Luebeck, Luebeck, Germany; ^2^Institute for Cardiogenetics, University of Luebeck, Luebeck, Germany; ^3^Department of Dermatology, University of Luebeck, Luebeck, Germany; ^4^Research Center Borstel, Leibniz-Center for Medicine and Bioscience, Borstel, Germany; ^5^Department of Dermatology, Venereology and Allergology, University Hospital Würzburg, Würzburg, Germany; ^6^The German Autoimmune Bullous Disease Genetic Study Group, Germany; ^7^Department of Dermatology, Venereology and Allergology, University Hospital Schleswig-Holstein, Kiel, Germany; ^8^Department of Dermatology, University Hospital of Dresden, Dresden, Germany; ^9^Department of Dermatology and Allergology, Phillips-Universität Marburg, Marburg, Germany; ^10^Institute of Medical Biometry and Statistics, University of Luebeck, Luebeck, Germany; ^11^Department of Dermatology, Faculty of Medicine, Medical Center-University of Freiburg, University of Freiburg, Freiburg, Germany; ^12^Dermatology Department, Faculty of Medicine, Dentistry and Health Sciences, The Royal Melbourne Hospital, University of Melbourne, Melbourne, VIC, Australia; ^13^Institute of Epidemiology, Christian-Albrecht University of Kiel, Kiel, Germany; ^14^Popgen Biobank, Christian-Albrecht University of Kiel, Kiel, Germany; ^15^Department of Dermatology, Venereology and Dermatooncology, Semmelweis University, Budapest, Hungary; ^16^Department of Dermatology and Allergy, University Hospital, LMU Munich, Munich, Germany; ^17^Department of Dermatology, Venereology and Allergology, Allergy Center Charité, Charité Medical University Berlin, Berlin, Germany

**Keywords:** mitochondrial DNA, mitochondrial haplogroup, polymorphisms, autoimmune skin diseases, bullous pemphigoid, mitochondrial function, next generation sequencing

## Abstract

Bullous pemphigoid (BP) is the most prevalent autoimmune skin blistering disease and is characterized by the generation of autoantibodies against the hemidesmosomal proteins BP180 (type XVII collagen) and BP230. Most intriguingly, BP is distinct from other autoimmune diseases because it predominantly affects elderly individuals above the age of 75 years, raising the question why autoantibodies and the clinical lesions of BP emerges mostly in this later stage of life, even in individuals harboring known putative BP-associated germline gene variants. The mitochondrial genome (mtDNA) is a potential candidate to provide additional insights into the BP etiology; however, the mtDNA has not been extensively explored to date. Therefore, we sequenced the whole mtDNA of German BP patients (*n* = 180) and age- and sex-matched healthy controls (*n* = 188) using next generation sequencing (NGS) technology, followed by the replication study using Sanger sequencing of an additional independent BP (*n* = 89) and control cohort (*n* = 104). While the BP and control groups showed comparable mitochondrial haplogroup distributions, the haplogroup T exhibited a tendency of higher frequency in BP patients suffering from neurodegenerative diseases (ND) compared to BP patients without ND (50%; 3 in 6 BP with haplogroup T). A total of four single nucleotide polymorphisms (SNPs) in the mtDNA, namely, m.16263T>C, m.16051A>G, and m.16162A>G in the D-loop region of the mtDNA, and m.11914G>A in the mitochondrially encoded NADH:ubiquinone oxidoreductase core subunit 4 gene (*MT-ND4*), were found to be significantly associated with BP based on the meta-analysis of our NGS data and the Sanger sequencing data (*p* = 0.0017, *p* = 0.0129, *p* = 0.0076, and *p* = 0.0132, respectively, Peto's test). More specifically, the three SNPs in the D-loop region were negatively, and the SNP in the *MT-ND4* gene was positively associated with BP. Our study is the first to interrogate the whole mtDNA in BP patients and controls and to implicate multiple novel mtDNA variants in disease susceptibility. Studies using larger cohorts and more diverse populations are warranted to explore the functional consequences of the mtDNA variants identified in this study on immune and skin cells to understand their contributions to BP pathology.

## Introduction

A number of studies to identify candidate genes in autoimmune blistering skin diseases, particularly bullous pemphigoid (BP) have been conducted to date. These studies predominantly identified associated gene polymorphisms in immune system-related genes, e.g., HLA region (1–6), Fc gamma receptor genes (7), and cytokine genes (8). In addition to these nuclear-encoded genes, our group has recently shown that polymorphisms in a gene encoded in the mitochondrial genome, *MT-ATP8*, are associated with BP (9). Considering the nature of autoimmune diseases, the causal factors of BP are not only limited to genetics, but also involve environmental factors. For example, our recent findings showed that the composition of skin microbiota was altered in BP patients compared to healthy controls (10).

The mitochondrial genome (mtDNA) is a circular DNA molecule with a length of ~16 kilobase pairs. The mtDNA encodes 13 protein-coding genes, 22 transfer RNA genes, and 2 ribosomal RNA genes (11, 12). Multiple copies of the mtDNA exist in a single mitochondrion. All of the 13 mtDNA-encoded proteins consist of subunits of the oxidative phosphorylation (OXPHOS) complexes, which are responsible for cellular energy production in the form of ATP, as well as for the production of reactive oxygen species (ROS) as a by-product of the OXPHOS reaction. The mtDNA is polymorphic, and variations in the mtDNA are known to be associated with alterations in mitochondrial functions (13). The mtDNA variations in humans are categorized into the following three groups: (1) recent maternally inherited deleterious mutations; (2) ancient adaptive polymorphisms; and (3) somatic mutations that accumulate during development and in tissues with age (14). Recent maternally inherited deleterious mtDNA mutations have been well-described in rare mitochondrial disorders, including familial mitochondrial encephalomyopathy (15) and Leber's hereditary optic neuritis (16). Ancient adaptive polymorphisms are commonly used to establish haplogroup ancestry, as these variations are believed to have occurred during the migration of human ancestors in order to adapt different environments (e.g., nutritional availability and climates) (17). Furthermore, several ancient polymorphisms in the mtDNA have been reported to be associated with common diseases, including chronic inflammation and autoimmune diseases (18, 19). These are not surprising because the fate and the function of immune cells are largely determined by cellular metabolism (immunometabolism), which is to a large extent controlled by mitochondrial functions (20–22). One example of such immune cell types is regulatory T cells, which have been reported to be involved in the pathology of BP (23–25), and their differentiation is determined by the levels of fatty acid synthesis, one of the mitochondrial functions (26). Longevity and aging have also been associated with certain mtDNA polymorphisms (27–29). Another unique characteristic of the mtDNA is the higher frequency of somatic mutations in aging compared to the nuclear genome (30), indicating the presence of variations identified only in elderly people. In fact, BP is the most prevalent autoimmune blistering skin disease and predominantly affects the elderly population, i.e., usually in late 70s (31, 32). As mentioned above, our group has recently demonstrated changes in the skin microbiota composition having been observed in BP patients compared to healthy controls (10). Recently, certain mtDNA haplogroups have been reported to be associated with the abundance of certain bacterial taxa (33). Consistent with these findings, our group recently identified that variations in the mtDNA are associated with the composition of microbiota in the gut (34) and the skin of mice (unpublished), suggesting that the variations in the mtDNA observed in BP patients contribute to a shift of the skin microbiota composition, which in turn enhances susceptibility to the disease. All of the abovementioned characteristics of the mtDNA support its potential involvement in BP.

Therefore, we have explored here the whole mitochondrial genome by next generation sequencing technology in German BP patients and their age- and sex-matched controls.

## Materials and Methods

### Study Cohorts

DNA samples for the NGS discovery study and the Sanger sequencing replication study were obtained from the German AIBD Genetics Study Group and PopGen Biobank. BP patients were diagnosed by clinicians at the participating centers. All included patients satisfied all of the following criteria; (i) a compatible clinical presentation, (ii) the detection of linear deposits of IgG and/or C3 at the dermal-epidermal junction by direct immunofluorescence (IF) microscopy of a perilesional skin biopsy, and (iii) the detection of serum autoantibodies against BP180 NC16A and/or BP230 by ELISA, according to the guideline of the German Dermatological Society for the diagnosis of BP (35). DNA samples from a total of 270 BP patients (180 for the NGS and 90 for a replication study using Sanger sequencing) and 294 controls (188 for the NGS and 106 for Sanger sequencing) were used in this study. The description of the cohort is summarized in Supplementary Table 1.

Of the samples tested by the NGS, 180 BP patients and 144 controls were evaluated for their genetic ancestry using their genome-wide SNP data, which were previously obtained using Affymetrix Biobank Axiom Array^TM^ (Thermo Fisher Scientific, MA, USA). Principal components analysis (PCA) (Supplementary Figure 1) plots of the 180 BP and 144 control samples almost overlapped, suggesting that the BP patients and controls in this study belonged to the same population.

Of the 180 BP patients whose mtDNA was sequenced, clinical history of neurodegenerative disease (dementia and Parkinson's disease) was available in 58 patients. Of the 58 BP patients, data on their anti-BP180 NC16A IgG titer were available in 49 patients.

The study was approved by the ethical committees of the University of Lübeck (10-026 and 15-051) and the individual study centers.

### Next Generation Sequencing of the Whole Mitochondrial Genome

Genomic DNA samples were processed for library preparation, as previously described in the Human mtDNA Genome protocol for Illumina Sequencing Platform (36). In brief, two primer sets [MTL-F1 (AAAGCACATACCAAGGCCAC) and MTL-R1 (TTGGCTCTCCTTGCAAAGTT); MTL-F2 (TATCCGCCATCCCATACATT) and MTL-R2 (AATGTTGAGCCGTAGATGCC)] were used to amplify the mtDNA by long-range PCR. Library preparation was performed using a Nextera XT DNA Library Preparation Kit (Illumina Inc., CA, USA), and the 10-pM library was sequenced on the Illumina MiSeq sequencing platform (2 × 150 bp paired-end reads) (Illumina Inc.).

### NGS Data Analysis

Our previously described data analysis method (37) was modified and adapted for human mtDNA analysis. After quality control, the reads were mapped to the revised Cambridge Reference Sequence (rCRS; NC_012920.1) using Burrows-Wheeler Aligner bwa version 0.705 (38), and bam files were generated. Duplicated reads generated during PCR were removed using Markduplicates (Picard tools version 1.119) (39), and indels were realigned using IndelRealigner (Genome analysis tool kit version 3.3) (40). The processed bam files were assessed for frequency and base quality (≥30) for each reference and alternate base in the mtDNA using pysamstats (version 0.24.3) (41). When the frequency of the alternate allele compared with the reference allele was >90%, it was considered as homoplasmic mutation, whereas the 10–90% range was considered as heteroplasmy. Additionally, bam files were manually inspected for the presence of mutations and indels using IGV software (42). mtDNA variants were annotated using MSeqDR mvTool (43), a DNA Web resource for comprehensive variant annotation.

### Mitochondrial Haplogroup Analysis

Mitochondrial haplogroup assignment was conducted using HaploGrep 2 (44). In brief, HaploGrep weighs each polymorphism present in PhyloTree17 (45) based on its informativeness to define haplogroups. The set of SNPs in the input file were classified as informative or remaining (not informative). A score is given based on the weights of the informative SNPs, and the offset was determined based on the number of remaining SNPs.

### Replication Study (Sanger Sequencing)

DNA samples for the replication study were prepared using standard DNA extraction kits (Qiagen, Hilden, Germany). SNP regions were amplified by standard PCR. The primers used for the PCR reaction are listed in Supplementary Table 2. The PCR products were sent to Genewiz (Essex, UK) for Sanger sequencing, and the obtained data were analyzed using the freely available software Unipro UGENE (46).

### Statistical Analysis

Data from the mtSNP association study and the mitochondrial haplogroup association study were analyzed using R package “exact2 × 2,” which provides a non-central confidence interval matching the two-sided Fisher's exact test based on the principle of likelihood estimation (47).

The meta-analysis was conducted using Peto's method (48) from the R package “metaphor” (49). This method provides a weighted estimate of the log odds ratio under a fixed-effects model. We used Hommel's method (50) to give strong control of the family-wise error rate, i.e., the probability of at least one type I error, by adjusting each *p*-value obtained from Peto's method.

Statistical analyses for other studies were performed using GraphPad Prism 6 (GraphPad Software, San Diego, CA, USA). Statistical tests used for the analysis are indicated in the figure legends.

## Results

### BP Patients in the Mitochondrial Haplogroup T May Exhibit a Higher Co-incidence With Neurodegenerative Conditions

**Figure 1:**
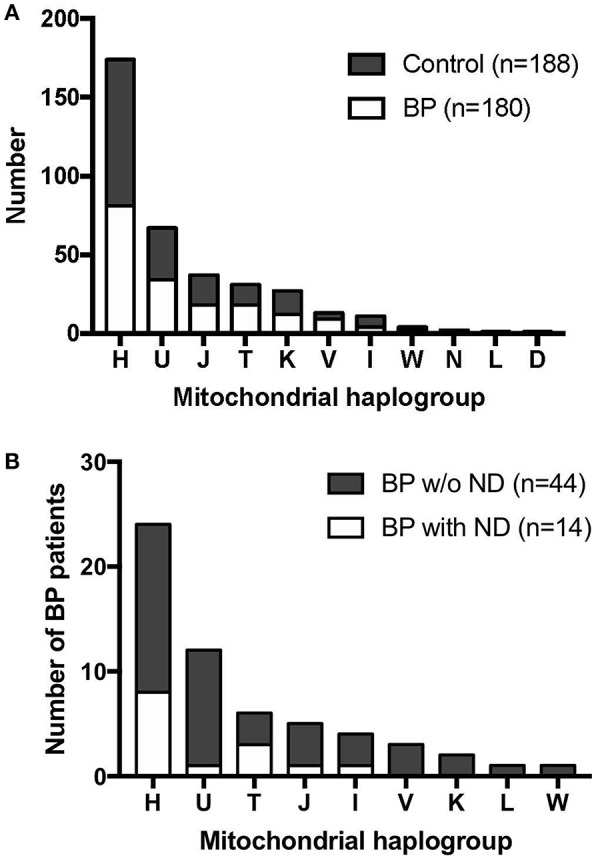
Mitochondrial haplogroup distribution in BP patients and controls. **(A)** BP patients and controls showed similar mitochondrial haplogroup distributions. **(B)** Mitochondrial haplogroup distribution when BP patients were stratified for clinical history of neurodegenerative diseases (ND). BP patients with the haplogroup T background tended to have higher incidence of ND.

Mitochondrial haplogroup analysis in this German population revealed that 47.28% of the sequenced individuals belonged to the haplogroup H, which is the major haplogroup in Europeans, followed by haplogroup U (18.21%), haplogroup J (10.05%), and haplogroup T (8.42%). When the data were analyzed for disease association, there was no association between BP status and the mtDNA haplogroups (*p* = 0.7963, Fisher's exact test, Figure 1A, Supplementary Table 3).

**Figure 2:**
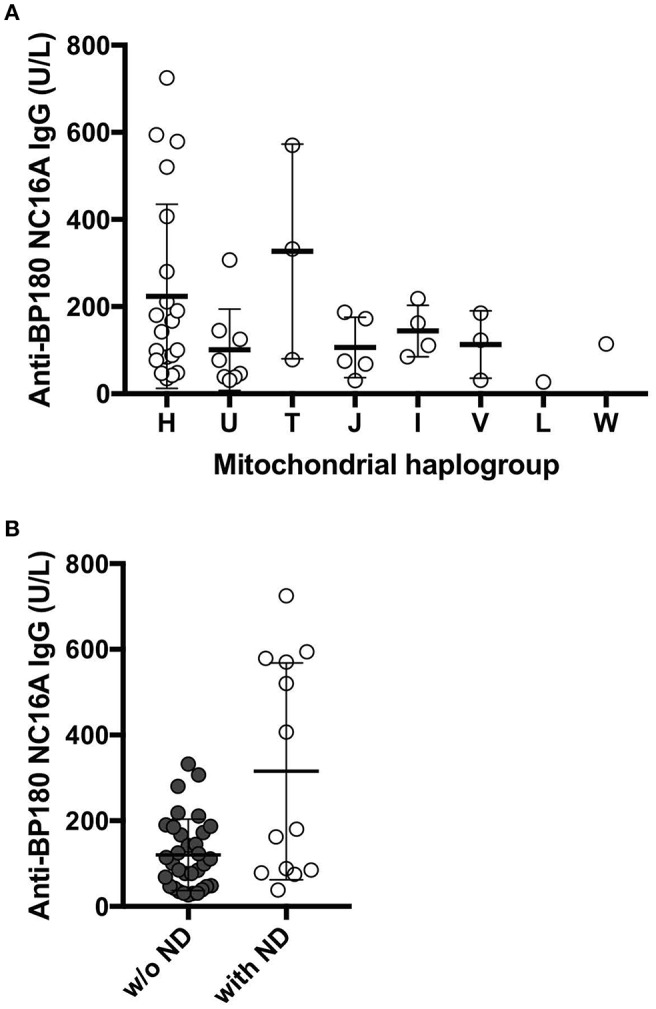
**(A)** The levels of anti-BP180 antibodies in individuals with each haplogroup. The mean values of anti-BP180 IgG were 223.8 U/L in haplogroup H, 101 U/L in haplogroup U, 326.7 U/L in haplogroup T, 106.4 U/L in haplogroup J, 144 U/L in haplogroup I, 113 U/L in haplogroup V, 27 U/L in haplogroup L, 114 U/L in haplogroup W. **(B)** The levels of the autoantibodies BP180 NC16A were highly varied in BP patients who also suffer from neurodegenerative diseases. *P* = 0.0272, Mann–Whitney test.

Among the 180 BP patients sequenced for the whole mtDNA, 58 cases had clinical history of neurodegenerative diseases (ND; e.g., Parkinson's disease and dementia), consistent with recent studies that reported an association between neurodegenerative diseases and BP in different populations (51). We analyzed the mitochondrial haplogroup associations in these 58 BP patients and found that BP patients in the mitochondrial haplogroup T tend to have higher risk of ND (Figure 1B, *p* = 0.1448, Fisher's exact test). We additionally evaluated 49 BP patients with available records of autoantibody (anti-BP180 NC16A IgG) levels. While no associations between the autoantibody titers and mitochondrial haplogroups were observed (Figure 2A), BP patients with concurrent ND showed significantly higher variation in autoantibody titers compared to BP patients without ND (Figure 2B, *p* = 0.027, Mann–Whitney test).

### Novel Candidate SNPs in the mtDNA-Associated With BP in Germans

A total of 1,010 SNPs in the mtDNA (mtSNPs) were identified in this study. The mtSNPs associated with BP (exploratory *p* < 0.1) are listed in Table 1. Of the five listed variants, the three mtSNPs; m.16263T>C, m.11914G>A, and m.15904C>T; were selected for the replication study using Sanger sequencing in the replication cohort of an additional independent 90 BP and 106 control samples. In this replication study, two relevant mtDNA sequences covering all three mtSNPs were PCR-amplified (Supplementary Table 2) and processed for Sanger sequencing. The Sanger sequencing data confirmed the validity of the NGS results obtained from the individuals carrying a variant from each of the three mtSNPs. In addition to the analysis of the three targeted SNPs, the design of the primers used for the Sanger sequencing replication study enabled us to evaluate the sequencing data of the locus ranging from m.15800 to m.16290 (covering a part of the displacement-loop region; D-loop region), as well as the locus ranging from m.11870 to m.12137 (covering a part of the mitochondrially encoded NADH:ubiquinone oxidoreductase core subunit 4 gene; *MT-ND4*) in the replication cohorts.

**Table 1:** Five candidate BP-associated SNPs in the mtDNA identified by the next-generation sequencing were selected for the replication study.

**mtSNP**	**Gene/region**	**Consequence**	**Homoplasmy**	**Heteroplasmy**	**Variant carrier in 180 BP**	**Variant carrier in 188 controls**	*p*-value^*^	**OR (95% confidence interval)**
m.9150A>G	*MT-ATP6*	Synonymous	+		0	9	0.0036	0.000 (0.000–0.491)
m.16263T>C	D-Loop	Non-coding	+	+	1	9	0.0201	0.112 (0.005–0.747)
m.13966A>G	*MT-ND5*	Non-synonymous	+		4	0	0.0563	∞ (0.945–∞)
m.11914G>A	*MT-ND4*	Synonymous	+		6	1	0.0626	6.422 (0.868–146.876)
m.15904C>T	*MT-TT*	Non-coding	+		9	3	0.0813	3.236 (0.876–14.143)

* *Two-sided p-value from Fisher's exact test: SNPs with explorative p-values <0.1 were selected for the replication study using Sanger sequencing*.

*mtSNP, single nucleotide polymorphism in the mitochondrial genome; BP, bullous pemphigoid; OR, odds ratio; CI, confidence interval*.

The meta-analysis of the NGS and the Sanger sequencing data revealed that m.16263T>C, m.11914G>A, m.16051A>G, and m.16162A>G were significantly associated with BP in Germans. Variants in the three mtSNPs in the D-loop region were more frequent in controls, while the variant in the m.11914G>A in the *MT-ND4* gene was more frequent in BP (Table 2). Meta-analysis revealed no significant association between m.15904C>T and BP (*p* = 0.2629, Peto's test).

**Table 2:** Meta-analysis of NGS data and Sanger sequencing data of BP patients and controls.

**mtSNP**	**Gene/region**	**Sequencing method**	**Group**	**Sequenced (*n*)**	**Variant carrier (*n*)**	**Frequency (%)**	**Odds ratio (95% CI)**	*P*-value*	**Q (*p* adjust, Hommel)**
m.16263 T>C	D-loop	NGS	BP	180	1	0.56	0.1116 (0.0051–0.7471)	0.0201	
			Control	188	9	4.79			
		Sanger	BP	82	0	0.00	0 (0–1.2049)	0.1222	
			Control	90	4	4.44			
		Meta-analysis	BP	262	1	0.38	0.1833 (0.0635–0.5293)	0.0017	0.0085
			Control	278	13	4.68			
m.11914G>A	*MT-ND4*	NGS	BP	180	6	3.33	6.4216 (0.8682–146.8755)	0.0626	
			Control	188	1	0.53			
		Sanger	BP	89	4	4.49	4.7190 (0.6014-−116.3248)	0.1860	
			Control	102	1	0.98			
		Meta-analysis	BP	269	10	3.72	4.2418 (1.3521–13.3077)	0.0132	0.0264
			Control	290	2	0.69			
m.15904C>T	*MT-TT*	NGS	BP	180	9	5.00	3.2360 (0.876–14.143)	0.0813	
			Control	188	3	1.60			
		Sanger	BP	88	4	4.55	0.7867 (0.2019–3.0665)	0.7574	
			Control	104	6	5.77			
		Meta-analysis	BP	268	13	4.85	1.6283 (0.6935–3.8232)	0.2629	0.2629
			Control	292	9	3.08			
m.16051A>G	D-loop	NGS	BP	180	2	1.11	0.6936 (0.0852–4.5071)	1	
			Control	188	3	1.60			
		Sanger	BP	88	0	0.00	0 (0–0.5505)	0.0041	
			Control	104	9	8.65			
		Meta-analysis	BP	268	2	0.75	0.2583 (0.0889–0.7505)	0.0129	0.0258
			Control	292	12	4.11			
m.16162 A>G	D-loop	NGS	BP	180	2	1.11	0.4121 (0.0574–2.0169)	0.4494	
			Control	188	5	2.66			
		Sanger	BP	88	0	0.00	0 (0–0.6679)	0.0081	
			Control	104	8	7.69			
		Meta-analysis	BP	268	2	0.75	0.2464 (0.0881–0.6889)	0.0076	0.0198
			Control	292	13	4.45			

*For the Sanger sequencing, 106 controls (for the genotyping of m.15904C>T, m.16051A>G, m.16162A>G, and m.16263T>C) or 104 controls (for the genotyping of m.11914G>A) and 90 BP samples were tested. The number of samples with no genotyping data due to the low quality-sequence was subtracted from the tested sample number*.

## Discussion

In this study, we performed NGS of the whole mitochondrial genomes of 180 BP patients and 188 controls, followed by a replication study using Sanger sequencing of independent samples of up to 90 BP patients and 104 controls from Germany. With cohorts tested in this study, we estimated the power of our study to detect mitochondrial variants. For the NGS study to detect SNPs to forward to replication, we used threshold of alpha = 0.1. Based on this, we estimated that for variants with frequencies of about 5% in controls, odds ratios of about 2.6 and higher would be detectable at a power of 80%; if the variants are more frequent with about 10% in controls, odds ratios of about 2.2 and higher are detectable reliability. For the replication study, Sanger sequencing on 90 independent cases and 104 independent controls was performed; applying a replication significance level of 0.05, this study was well-powered with a power of at least 80% to detect odds ratios of at least 4.2. This might explain why we were not able to replicate the variant m.15904C>T because the power for this SNP might have been too low. The study cohort is the largest for this disease worldwide, however, it is still relatively small in size compared to those used in genetic studies of other chronic inflammatory skin diseases, e.g., psoriasis or atopic dermatitis. This is primarily due to the rarity of the disease. Nevertheless, this study is highly valuable to the field as it is the first to interrogate the whole mitochondrial genome by sequencing in autoimmune blistering skin diseases.

The whole mtDNA sequencing data were analyzed for mitochondrial haplogroup associations with BP. As mentioned above, mitochondrial haplogroups are defined by specific combinations of ancient adaptive polymorphisms in the mtDNA and often reflect on mitochondrial functionality to adapt to the specific environments according to the geographical locations where our ancestors migrated. Therefore, mitochondrial haplogroups have been used to define ethnic origins on mostly prehistoric time scales. In fact, as previously shown (52), combinations of the adaptive polymorphisms in the mtDNA altered the mitochondrial functions, which are causal for common complex diseases in certain populations.

Our findings showed that the haplogroup distribution between BP and controls in this study was comparable. However, interestingly, when we correlated the mitochondrial haplogroup and the presence of neurodegenerative diseases (ND; i.e., dementia and Parkinson's disease) in BP patients, the analysis revealed that the haplogroup T appeared more frequent in BP patients suffering from ND comorbidity. Haplogroup T is known to be associated with ND (53, 54). In parallel, the levels of anti-BP180 antibodies in individuals with haplogroup T exhibited a tendency of higher levels though the result is exploratory (average values of 326.7 U/L in individuals with haplogroup T, while the average values in all BP patients were 175.6 U/L). Together with the positive association between the levels of anti-BP180 antibodies and the presence of ND, the link between mitochondrial haplogroup, neurodegenerative disease and the levels of anti-BP180 autoantibodies among BP patients may be plausible. The haplogroup J, in contrast, did not share the same tendency, even though haplogroup J and T belong to the same sub-cradle of the haplogroup JT. These results are in agreement with those of previous studies demonstrating that the haplogroup J is protective against Parkinson's disease (18, 55), which suggested that the mtDNA variants defining the haplogroup J may be protective from concurrent ND in BP patients. Furthermore, the average levels of autoantibodies in individuals with haplogroup J were 106.4 U/L, which were lower than the average values of all BP patients in this analysis. Nevertheless, to confirm this exploratory observation, further studies should analyze a larger patient cohort with available clinical histories.

Next, whole mtDNA sequencing data were analyzed for associations between the single nucleotide polymorphisms in the mtDNA (mtSNPs) and BP. The analysis identified five top candidate mtSNPs. The meta-analysis of the discovery study (whole mtDNA NGS data) and the replication study (Sanger sequencing data of partial mtDNA region) revealed four mtSNPs that were significantly associated with BP. The three mtSNPs located in the non-coding D-loop region, namely, m.16051A>G, m.16162A>G, and m.16263T>C, were all enriched in the controls, while the mtSNP m11914 in the *MT-ND4* gene was more frequent in BP patients. The D-loop is a non-coding region of the mtDNA, including the replication origin of the H (heavy) strand (OriH), the promoters for transcription of the H and L (light) strand (HSP and LSP) and two hyper variable segments (HVS1: m.16,024-m.16,383, and HVS2: m.57-m.372), constituting the most variable regions in the mtDNA (56). All three mtSNPs in the D-loop region are located in HVS1. Functional consequences of the variants in this non-coding D-loop region, particularly the HVS, remain unknown to date. Interestingly, most carriers of a variant from any of these three mtSNPs in the D-loop region belong to haplogroup H; two of the 81 BP patients belonged to haplogroup H, and 14 of 93 controls belonged to haplogroup H. Given that the three mtSNPs serve as defining SNPs for haplogroup H-subgroups, i.e., m.16051A>G for H1a3 and H2a2a1c (45), the functional relevance of these mtSNPs may be linked with other haplogroup H-subgroups-defining SNPs. Considering the limited sample size of patients with a rare disease, a more finely-branched subgroup haplogroup analysis cannot be conducted in this study. This also applies to the mtSNP m.11914G>A. The A variant enriched in the BP group is a synonymous mutation in the *MT-ND4* gene, suggesting that its functional relevance is unlikely. The variant in m.11914G>A is also a defining SNP for several haplogroups-subgroups U, K, T, and H (45). Three of 12 BP patients and one of 15 controls belonging to haplogroup K carried the A variant in m.11914G>A.

Previously, our group has reported an association between a rare variant in the *MT-ATP8* gene and BP in Germans (9). The current study is an extension of the previous study to explore the whole mtDNA in a German cohort, including other individuals than those enrolled in the previous study. The BP-associated mtSNP, m.8519G>A, which was identified in our previous study, was found in one BP patient among newly sequenced 137 BP patients and 20 controls in the current study (*p* = 1.000, Fisher's exact test). The meta-analysis of the NGS data and the previously published Sanger sequencing data still showed a positive association between m.8519G>A and BP (*p* = 0.0151, Peto's test, odds ratio 7.3454, 95% confidence interval 1.4718–36.6594).

We utilized peripheral blood DNA samples in this study. Somatic mutations accumulate over time in tissues with age (17), and the levels of the mutant mtDNA, i.e., levels of heteroplasmy, can be different between various tissues and organs (30, 57). Therefore, an evaluation of the whole mtDNA sequence in other tissues of importance, i.e., skin samples obtained from BP patients and controls, is warranted. Within BP skin samples, site different samples, i.e., peri-lesional and unaffected, are also of great interest to evaluate differential levels of the mtDNA mutations within the same individual. Such age-dependent and tissue-specific changes in the mtDNA may elucidate the pathways in late-onset diseases such as BP. Thus, the interpretation of the results in this study needs to be cautious as the results obtained from this study using peripheral blood DNA might not be the same as those using tissue DNA. As aforementioned, BP is a multifactorial disease, and the involvement of immune cells and skin microbiota in the disease has been proposed (10, 58). Both the immune system and the composition of microbiota are altered in aging (59–62), interact with each other (63), and are associated with mitochondrial functions (21, 64). Complex interactions between these age-related alterations and age- and tissue-specific mtDNA variants could provide key insights to the pathways in diseases with complex traits. To date, only a few studies using mice proposed an association between mtDNA variants, microbiota and clinical phenotypes have been reported (34, 65), but none in humans.

In summary, we investigated genetic variants in the whole mtDNA genome in German BP patients and their age- and sex-matched controls, which is currently the largest available study cohort for BP worldwide. Our findings showed that the maternally inherited natural variants in the mtDNA are associated with BP, which predominantly affects the elderly population. Therefore, more complex interactions between nuclear genome variants and mtDNA variants, as well as aging, are likely to be involved in the pathogenesis of BP. To identify the functionally relevant mtDNA variants in BP, studies with the larger sample sizes and analysis of the mtDNA genome in the skin should be conducted.

## Data Availability

Sequencing data used for this study is available under controlled access from the European Genome-phenome Archive with the Study ID EGAS00001003932 (https://ega-archive.org/studies/EGAS00001003932).

## Ethics Statement

This study was conducted in accordance with the recommendations of institutional review board of the University of Lübeck, and the review boards of the collaborating centers. The protocol was approved by the institutional review board of the University of Lübeck (File No. 10-026 and 15-051), and the review boards of the collaborating centers. All subjects provided written informed consent in accordance with the Declaration of Helsinki. Samples and demographic data of patients and controls were collected in adherence to ethics and German privacy protection regulations.

## Author Contributions

JR, MWi, and JY performed the experiment. SB, RG, CG, RE, MMH, JK, DK, CS, MS, FS, NB, AW, MWo, and DZ recruited the patients and control cohorts. ES initiated and coordinated the German AIBD Genetics Study group. WL provided POPGEN control cohort DNA samples. AF, HB, JE, and YG performed the NGS data process. JR, AF, HB, DG, and IK conducted statistical analysis. JR, SI, and MH interpreted the data and wrote the manuscript with support of all co-authors. SI and MH designed the study.

### Conflict of Interest Statement

The authors declare that the research was conducted in the absence of any commercial or financial relationships that could be construed as a potential conflict of interest.
