# Supplementary material for: Polymorphisms in the Mitochondrial Genome Are Associated With Bullous Pemphigoid in Germans
Source: Front Immunol. 2019 Nov 22;10:2200. doi: 10.3389/fimmu.2019.02200 (PMC6883920; doi:10.3389/fimmu.2019.02200)
Supplement: Supplementary file 1 [file Data_Sheet_1.docx]

**Supplementary Material**

**Supplementary Figure 1**


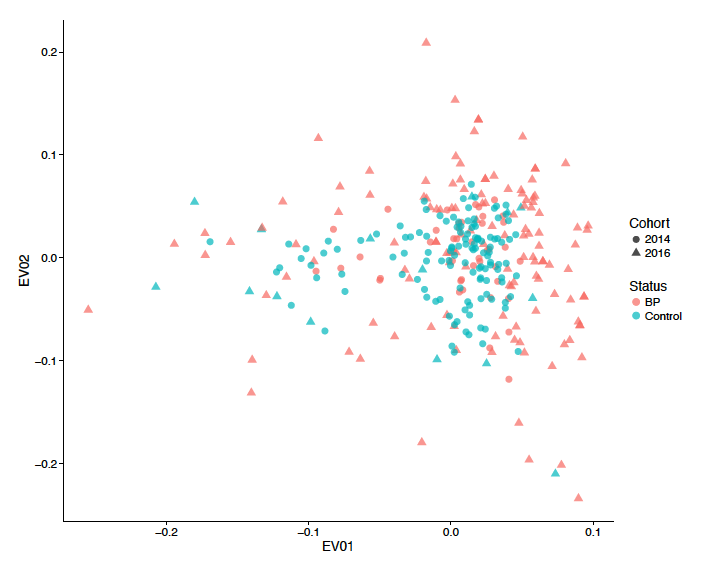


**Supplementary Figure 1: Genetic ancestry was similar in German cohorts tested in this study.** SNP data from GWAS study of a total of 180 BP and 144 controls tested for the whole mtDNA sequencing were evaluated for their ancestry genetic background. These samples were genotyped either in 2014 or 2016. Principal component analysis showed that the plots of BP and control samples clustered similarly, suggesting the ancestry genetic difference between BP and controls were negated, i.e., they are genetically Germans.

**Supplementary Table 1. Description of the cohort tested in this study.**

|  | NGS | | | | Sanger sequencing | | | | Total | | | |
| --- | --- | --- | --- | --- | --- | --- | --- | --- | --- | --- | --- | --- |
|  | Control | | BP | | Control | | BP | | Control | | BP | |
|  | N | Ave. age | N | Ave. age | N | Ave. age | N | Ave. age | N | Ave. age | N | Ave. age |
| Male | 92 | 76 | 81 | 79 | 51 | 78 | 51 | 78 | 143 | 76 | 132 | 79 |
| Female | 96 | 77 | 99 | 79 | 48 | 76 | 38 | 80 | 144 | 76 | 137 | 79 |
| Unknown | 0 |  | 0 |  | 7 | N.A. | 1 | N.A. | 7 | N.A. | 1 | N.A. |
| Total | 188 | 76 | 180 | 79 | 106 | 77 | 90 | 79 | 294 | 76 | 270 | 79 |

Age information was available from all control samples, 65 male BP and 72 female BP tested for the NGS study. For the samples tested for the Sanger sequencing, age information was available from all male controls, 46 female controls, 50 male BP and 36 female BP samples. N.A.; not available.

**Supplementary Table 2. Primers used for Sanger sequencing.**

| Target mtSNP | Primer name | Sequence 5'-3' | Product size (bp) |
| --- | --- | --- | --- |
| m.15904 C>T and m.16263 T>C | 15718_F | TTATTGACTCCTAGCCGCAGAC | 738 |
|  | 16455_R | CGGAGCGAGGAGAGTAGCAC |  |
| m.11914 G>A | 11763_F | AACGCACTCACAGTCGCAT | 492 |
|  | 12254_R | TGTTGTTAGACATGGGGGCAT |  |

**Supplementary Table 3. Mitochondrial haplogroup distribution in German population in this study.**

|  | Mitochondrial haplogroup | | | | | | | | | | |
| --- | --- | --- | --- | --- | --- | --- | --- | --- | --- | --- | --- |
|  | H | U | J | T | K | V | I | W | N | L | D |
| BP (n=180) | 81 | 34 | 18 | 18 | 12 | 9 | 4 | 2 | 1 | 1 | 0 |
| Control (n=188) | 93 | 33 | 19 | 13 | 15 | 4 | 7 | 2 | 1 | 0 | 1 |
